# Rethinking Primary Care Delivery Models: Can Integrated Primary Care Teams Improve Care Experience?

**DOI:** 10.5334/ijic.5945

**Published:** 2022-04-27

**Authors:** Arnaud Duhoux, Émilie Dufour, Martin Sasseville, Dominique Laroche, Damien Contandriopoulos

**Affiliations:** 1 Faculty of nursing, University of Montreal, CA; 2 Charles-Le Moyne Research Center, CA; 3 CISSS de Laval’s Research center on transformation of clinical and organisational practices, CA; 4 Faculty of Medicine, University of Sherbrooke, CA; 5 School of Nursing, University of Victoria, CA

**Keywords:** community care, interdisciplinary teams, delivery of health care, patient-reported measures

## Abstract

**Background:**

Integrated Primary Care Teams (IPCTs) have four key characteristics (intensive interdisciplinary practice; advanced nursing practice with an expanded role; group practice; increased proximity and availability) aimed at strengthening primary care in Quebec, Canada. The purpose of this paper is to examine the care experience over time of patients who have an IPCT as their primary source of care.

**Methods:**

We used a quasi-experimental longitudinal design based on a pre-and-post administered survey at a 2-year interval without a control group. We measured patient-reported accessibility, continuity, comprehensiveness, responsiveness and outcomes of care.

**Results:**

Results showed that patients who were newly registered with an IPCT had a significant increase in reported care experience, whereas patients who have been registered with an IPCT for 2 years prior to the first round of data collection had already high reported care experience that was maintained over time. Moreover, linear regression models showed statistically significant different increases in the dimensions of care experience by site and patients’ characteristics.

**Conclusions:**

Our results suggest that the IPCT model is tailored to the needs of its target populations, resulting in improved Patient Reported Experience Measures. These results imply that broader implementation of innovative and flexible community-based care models should be considered by policymakers.

## Background

Most jurisdictions worldwide are trying to identify ways to strengthen their primary care capacities in ways that can lead to performance improvements at the system level [1,2,3,4,5,6,7]. Along the way, it is getting increasingly clear that the solutions go beyond providing more of the same [8]. Primary care strengthening should include redefining the nature of the care provided as well as the professional roles and task sharing within teams [5,9,10]. One perspective for strengthening primary care is that of interprofessionalism, defined as an approach where care is provided by teams composed of a majority of non-physician clinicians [11].

This interprofessionalism is often achieved through an increase of the scope of nursing practice, which is likely to improve accessibility of care and efficiency of delivery [12]. However, although interdisciplinary teams incorporating an enhanced nursing role have strong potential, there are many practical challenges [9,10,13,14,15,16,17,18,19,20,21]. Such an approach involves redefining professional boundaries and revisiting existing care models and organisational arrangements.

This paper is part of a larger project whose full research protocol has been published elsewhere [22]. The findings are based on the study of six primary care teams in Quebec (Canada) identified as Integrated Primary Care Teams (IPCTs). The teams were identified through preliminary interviews with stakeholders. Four inclusion criteria were used to define them: 1) an extensively interdisciplinary practice where at least half of the professionals are nonphysicians professionals (nurses, PCNPs, nutritionists, social workers, etc,); 2) an advanced nursing practice with an expanded nursing scope of practice; 3) a group-based practice where the team shares resources and accountability; 4) the increased proximity and availability of care [22]. These four characteristics make them significantly different from the usual primary care structures in Quebec.

Quality of primary care services is typically assessed through two main sources of data. On one hand, administrative databases mostly focus on physicians billing, prescription drugs and hospital use. On the other hand, Patient Reported Outcome Measures (PROM) and Patient Reported Experience Measures (PREM) provide patient-centered measures. This paper focuses on PREM, while the larger IPCT study integrates both sources of data. The main strength of PREM is to allow for the capture of quality dimensions that are important to patients but that are not captured by administrative data. The aim of this paper is to examine the care experience over time of patients who have an IPCT as their primary source of care. The specific objectives are:

1. to examine if the experience of care, in its five studied dimensions, changed over a period of two years among these patients;
2. to examine which IPCT as well as patient’s individual characteristics are associated with an improved care experience.

## Methods

### Research design

The general aim of the IPCT project is to gain a better understanding of the relation between the aforementioned four characteristics of these primary care teams and their effects [22]. The data presented in this paper are part of the quantitative evaluation of these effects. We used a quasi-experimental longitudinal design without a control group based on a pre and post survey of patients whose primary source of care was an IPCT to analyse their experience of care.

### Recruitment of IPCT and patients and data collection

The starting point of our recruitment strategy is a convenience sample of six IPCTs located in the province of Quebec (Canada). All IPCTs provide care within either urban (Montreal or Quebec City) or dense suburban settings (Montreal South Shore). As mentioned previously, these IPCTs are deliberately not representative of the average primary care team in Quebec, as the inclusion criteria was to find teams which are more interprofessional and more innovative than the average. Primary care teams meeting these criteria were rare at the start of our study.

The six IPCTs differ in terms of their populations of interest and mission, their size and their team composition (***Table 1***). Three of them (IPCTs A, B and C) are intended to provide general care to an overall population and therefore have a structure similar to that of the common primary care practice model, the Family Practice Groups (FMGs). The three other IPCTs (D, E and F) involve vulnerable populations, including immigrant and homeless populations. Their focus is to provide a range of care and services that address the needs of these populations. IPCTs E also aim to promote the reintegration of patients into the health care system. The IPCT B doesn’t officially fulfil the first inclusion criteria, however it has developed a service agreement with a public clinic that is in the same building to have access to several services, such as social workers, registered nurses or respiratory therapists, which allows this IPCT to meet this criterion.

**Table 1:** Characteristics of IPCTs.


	IPCT A	IPCT B	IPCT C	IPCT D	IPCT E	IPCT F

**Composition of the care team (Full-time equivalent)** ^1^	MD = 10 NP = 2 RN = 5,5 LPN = 1,5 SW = 0,5 Psychologist = 0,6	MD = 5 RN = 2,6	MD = 8 NP = 1 RN = 6 Other = 3	MD = 11,5 NP = 3 RN = 10,5 SW = 1 Other = 2	MD = 0,6 RN = 1 Peer helpers = 0,8	NP = 1,5 RN = 1 SW = 0,3

**Type of clientele**	General population	General population	General population	General population and recent immigrants	Marginalized people^2^	General population and marginalized people^2^

**Number of registered patients at the time of the study**	10 000–15000	5000–10 000	10 000–15 000	5000–10 000	<2500	<2500


^1^ Medical Doctor (MD), Nurse Practitionner (NP), Registered nurse (RN), Licensed Practical Nurses (LPN), Social worker (SW). ^2^ Homeless people, drug users, people from prison or prostitution.

We recruited patients who used these IPCTs as their primary source of care in each setting. During randomly chosen periods to ensure proper representation of every day of the week and of different period of the day (morning, afternoon, and evening), all the patients were invited to participate to the study by trained interviewers in the waiting rooms of the IPCTs. Patients who agreed to participate were surveyed at two points in time (t0 and t2). The questionnaire for t0 was completed at the time of the recruitment in person on a tablet computer with the help of the trained interviewer if needed either in English or French. To allow patients from minorities to participate, a telephone translation service was used when needed. The patients were retrieved 2 years later to complete the questionnaire either by phone or online (t2). Two questionnaires were developed based on the history of use of the IPCTs at t0. A first questionnaire (Q1) was developed for patients for whom IPCT had been the primary source of care for less than 2 years, focusing on their experience before their first IPCT visit. The second questionnaire (Q2) was developed for patients for whom the IPCT was the regular source of care for at least 2 years at t0, focusing on their experience in that IPCT. As two of the IPCTs (IPCT E and IPCT F) were launched less than 2 years prior to the data collection, only Q1 questionnaires were administered at these sites. Both t0 and t2 questionnaires featured the same measuring tool to assess experience of care. The survey also included self-reported questions regarding age, sex, matrimonial status, perceived economic situation, occupation, chronic illness, education level and health status.

### Definition and Operationalization of the Variables

The conceptualization of patient’s experience of care is based on the work of Pineault [23] and defined as how the care and the services received are perceived or felt by individuals. Within this framework, experience of care is comprised of five attributes which can be evaluated by the patients: accessibility, continuity, comprehensiveness, responsiveness as well as outcomes of care (See ***Table 2*** for definitions). We used a questionnaire built by Pineault [24]. This questionnaire was adapted from two validated instruments, the Primary Care Assessment Survey and the Primary Care Assessment Tool, to which Pineault and colleagues added questions when a topic had not been addressed [25,26]. This 27-items questionnaire allows us to calculate 5 scores based on the 5 attributes of experience of care. Scores have a different number of items attributed, from 4 (Continuity) to 7 (Responsiveness). Each of the respondents’ response are indexed on scores ranging from 0 to 10, the highest score indicating the most positive care experience (see Appendix I). For every item, the “I don’t know/I don’t remember” responses were aggregated to the most neutral value [5], according to Haidar et al (2018) [27].

**Table 2:** Definitions of attributes of experience of care.


ATTRIBUTES	DEFINITIONS

Accessibility	The patient’s perception on the possibility to obtain healthcare services [23].

Continuity	The seamless flow in which multiple services are to be provided. These services are continuous if they are harmoniously linked to each other (management continuity) and when patients are treated continuously by the same professional or the same team (relational continuity) [23,28].

Comprehensiveness	How the patient perceives that all his needs for care are addressed [23].

Responsiveness	How the system responds to legitimate the expectations of the patient in regards to the non-technical elements or actions of a treatment [23].

Outcomes of care	The patient’s perception of the effects or the consequences of the received care on his health [23].


To examine which IPCT as well as patient’s individual characteristics are associated with an increase in care experience, we used the following variables: regular source of care (we constructed a dummy variable for each IPCT), sex, age, matrimonial status (married or law-partner/single, widowed or divorced), highest level of education completed (high school diploma or less/post-secondary degree), perceived economic situation (poor or very poor/sufficient income or financially comfortable), employment status (full, part time or self-employed/not employed, student or retired), chronic illness (reported diagnosis of high blood pressure, diabetes, asthma, chronic bronchitis, emphysema or chronic obstructive pulmonary disease, depression, anxiety, other mental health disorder). Health status was assessed using the Short Form 12-item health survey (SF-12), which assesses individual’s functioning within the previous four weeks of test administration [29]. The results are expressed in terms of a meta-score that reflects physical and mental health functioning: The Physical Component Summary (PCS) and the Mental Health Component Summary (MCS). Scores on the items are weighted to produce a range from 0 to 100, with scores higher than 50 indicating above average health status. The SF-12 has demonstrated sound psychometric properties [29,30].

### Data analysis

For the first objective, we conducted a descriptive analysis and proceeded to T tests on paired samples with a threshold of 0.05. When the sample was smaller than 30, a non-parametric Wilcoxon test for paired samples was used. For the second objective, we used multivariate linear regression models with forced entry to assess which variable is associated with an increase in experience of care between t0 and t2. We arbitrarily chose IPCT D as the reference for the analysis and we controlled for regular clinic frequented between t0 and t2 as well as scores from t0. The dependent variables used in this study represent the difference between t0 and t2 in the scores of the 5 attributes of experience of care. Due to missing data, sample size varies for each model. The statistical analyses were carried out using the SPSS software version 25.

### Ethics

The project has been accepted by the University of Montreal Health Sciences Research Ethics Committee (CERES, number 2014-488: CERES-14-141-P) and the Research Ethics Committee of the Centre de Santé et de Services Sociaux de la Montagne.

## Results

### Description of participants

Of the 2186 patients who were recruited in t0, 1473 (67.4%) took part in t2. Baseline characteristics of the sample are shown in Appendix II. Participants are mainly women (62.7%), aged 45 or older (58.2%), with a post-secondary degree (71.7%). They mainly consider themselves as “financially comfortable” or with a sufficient income (80.3%) and are in common-law relationship or married (63.4%). The participants who completed t2 are not statistically different from participants lost to follow-up between t0 and t2 on any studied variable. However, we can note large differences between the IPCTs. In particular, participants in IPCT E and F consider themselves more often as poor or very poor and report higher rates of mental illness.

### Evolution of the experience of care in its 5 dimensions

**Figure 1:**
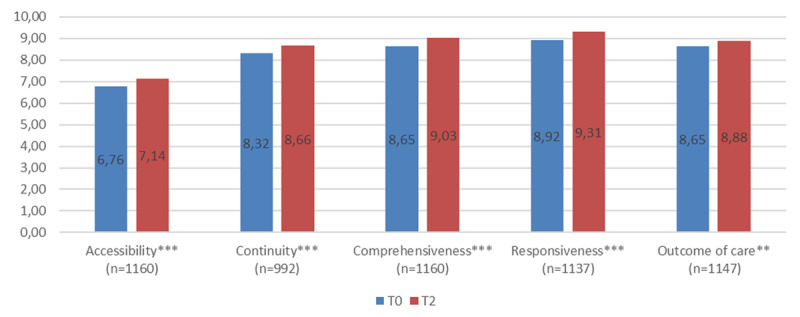
Assessment of care experience in 5 dimensions, t0 and t2, for 6 IPCT in Quebec. * P < 0.05; ** P < 0.01; *** P < 0,001.

The experience of care improved slightly (from 0,23 to 0,39 points) but significantly for the entire sample between t0 and t2 on all 5 dimensions (***Figure 1***). However, most of the IPCTs haven’t showed statistically significant improvement on some dimensions (***Table 3***). A score increase on 4 or 5 dimensions was observed for IPCT E and F, suggesting that the implementation of these IPCTs had a significant impact on the experience of care of their patients.

**Table 3:** Scores of care experience regarding 6 IPCTs for t0 and t2.


		ACCESSIBILITY		CONTINUITY		COMPREHENSIVENESS		RESPONSIVENESS		OUTCOME OF CARE	
				
		t0	t2	t0	t2	t0	t2	t0	t2	t0	t2

IPCT A	Q1	5,0	7,0**	6,5	8,4	5,8	8,0*	6,5	9,3***	5,7	8,4*

	Q2	7,1	6,9	8,4	8,7	9,2	9,2	9,4	9,4	8,9	8,8

IPCT B	Q1	6,3	7,7***	8,6	9,1	8,5	8,9	8,5	9,5***	8,5	9,1*

	Q2	7,8	7,7	8,5	8,7	9,1	8,8*	9,3	9,2	8,9	8,6*

IPCT C	Q1	5,9	7,0***	8,0	8,5	7,6	9,2***	8,2	9,4***	7,8	9,0***

	Q2	6,6	6,7	8,8	9,0**	9,3	9,3	9,2	9,2	9,2	9,2

IPCT D	Q1	5,8	6,0	7,3	7,6	6,8	8,7*	7,7	9,1**	7,5	8,2

	Q2	6,5	6,4	7,8	8,2	8,4	8,3	8,8	8,7	8,4	8,5

IPCT E	Q1	5,4	6,8**	7,8	7,5	7,3	8,9*	8,3	9,3**	8,1	8,4

IPCT F	Q1	5,5	8,0***	7,2	8,4**	7,1	9,3***	8,0	9,7***	7,5	9,0***


* P < 0.05; ** P < 0.01; *** P < 0,001.

We also observed that new patients (Q1) of other IPCTs had a significant increase of care experience compared to their previous regular source of care. Conversely, we observed that patients whom already had the IPCT as a regular source of care (Q2) had already high scores on each scale at t0. Hence, a low increase was observed at t2. For example, scores increased significantly from t0 to t2 in four of the five dimensions for patients who answered the Q1 questionnaire in IPCTs A and C, while we observed very little variation in the already high scores for Q2 patients between t0 and t2.

All IPCTs showed a significant increase in the dimension of responsiveness for patients who responded to Q1. Scores for this dimension were stable for patients responding to Q2. Having an IPCT as a primary source of care also appears to have an impact on accessibility and comprehensiveness as we observed significant increases in five of the IPCTs for patients who responded to Q1.

We observed that continuity scores were already high at t0 for most IPCTs, which may explain the significant increase in this dimension for a single site (IPCT F) in Q1. We also observed a slight but significant increase on one site (IPCT C) on Q2 for this same dimension.

### IPCTs and the individual characteristics associated with an increase in experience of care

Linear regression models showed statistically significant different increases in the dimensions of experience of care depending of the site compared with the reference site except for outcome of care (***Table 4***). The most significant difference is in accessibility where the IPCT F has a beta of 1,524 (CI95% (1.00; 2.03)).

**Table 4:** Multivariate linear regression models of the 5 dimensions of care experience for 6 IPCT in Quebec.


	ACCESSIBILITY (n = 1133)			CONTINUITY (n = 971)			COMPREHENSIVENESS (n = 1135)			RESPONSIVENESS (n = 1116)			OUTCOMES OF CARE (n = 1126)		
				
	B	SD	CI 95%	B	SD |	CI 95%	B	SD	CI 95%	B	SD	CI 95%	B	SD	CI 95%

Constant	2,824	0,456	(1.92, 3.71)	6,035	0,537	(4.98, 7.08)	5,129	0,506	(4.13, 6.12)	6,585	0,308	(5.98, 7.18)	5,754	0,488	(4.79, 6.71)

IPCT A	**0,580**	**0,211**	(**0.16, 0.99**)	0,393	0,255	(–0.10, 0.89)	0,463	0,239	(–0.00, 0.93)	**0,502**	**0,129**	(**0.24, 0.75**)	0,240	0,232	(–0.21, 0.69)

IPCT B	**0,997**	**0,206**	(**0.59, 1.40**)	0,462	0,244	(–0.01, 0.94)	0,133	**0,231**	(–0.31, 0.58)	**0,320**	**0,125**	(**0.07, 0.56**)	0,047	0,224	(–0.39, 0.48)

IPCT C	0,343	0,204	(–0.05, 0.74)	**0,627**	**0,243**	(**0.15, 1.10**)	**0,510**	0,231	(**0.05, 0.96**)	**0,298**	**0,125**	(**0.05, 0.54**)	0,433	0,224	(–0.00, 0.87)

IPCT E	0,577	0,336	(–0.08, 1.23)	–0,566	0,402	(–1.35, 0.22)	0,337	0,373	(–0.39, 1.06)	0,307	0,202	(–0.08, 0.70)	–0,245	0,358	(–0.94, 0.45)

IPCT F	**1,524**	**0,263**	(**1.00, 2.03**)	0,350	0,318	(–0.27, 0.97)	**0,768**	**0,291**	(**0.19, 1.33**)	**0,571**	**0,158**	(**0.26, 0.88**)	0,426	0,282	(–0.12, 0.97)

Gender (women)	0,028	0,098	(–0,16)–(0,22)	**–0,282**	**0,120**	(**–0.51, –0.04**)	**–0,245**	**0,109**	(**–0.45, –0.03**)	–0,043	0,059	(–0.15, 0.07)	**–0,245**	**0,104**	(**–0.44, –0.04)**

Age	0,001	0,003	(–0.00, 0.00)	0,006	0,004	(–0.00, 0.01)	0,001	0,003	(–0.00, 0.00)	**0,004**	**0,002**	(**0.00, 0.00**)	0,002	0,003	(–0.00, 0.00)

Married or law-parner	–0,128	0,103	(–0.33, 0.07)	–0,148	0,126	(–0.39, 0.09)	0,013	0,115	(–0.21, 0.23)	–0,005	0,062	(–0.12, 0.11)	0,149	0,110	(–0.06, 0.36)

Highest level of education – Diploma of college or less	**0,224**	**0,099**	(**0.02, 0.41**)	0,022	0,120	(–0.21, 0.25)	0,099	0,111	(–0.11, 0.31)	0,087	0,060	(–0.03, 0.20)	**0,254**	**0,106**	(**0.04, 0.46**)

Economic situation – very poor/poor	–0,246	0,141	(–0.52, 0.03)	–0,128	0,168	(–0.45, 0.20)	0,221	0,157	(–0.08, 0.52)	0,064	0,084	(–0.10, 0.22)	0,024	0,149	(–0.26, 0.31)

Employment status – full/part time/self- employed	–0,125	0,098	(–0.31, 0.06)	–0,154	0,118	(–0.38, 0.07)	0,020	0,109	(–0.19, 0.23)	0,024	0,059	(–0.09, 0.14)	–0,009	0,104	(–0.21, 0.19)

SF12 – Physical health	**0,013**	**0,004**	(**0.00, 0.02**)	–0,001	0,005	(–0.01, 0.00)	**0,011**	**0,005**	(**0.00, 0.02**)	0,004	0,003	(–0.00, 0.00)	0,001	**0,005**	(–0.00, 0.01)

SF12 – Mental health	**0,022**	**0,005**	(**0.01, 0.03**)	**0,014**	**0,005**	(**0.00, 0.02**)	**0,031**	**0,005**	(**0.02, 0.04**)	**0,017**	**0,003**	(**0.01, 0.02**)	**0,020**	0,005	(**0.01, 0.02**)

High blood pressure or hypertension	0,019	0,117	(–0.21, 0.24)	–0,205	0,138	(–0.47, 0.06)	–0,151	0,131	(–0.40, 0.10)	–0,131	0,071	(–0.26, 0.00)	–0,216	0,125	(–0.46, 0.02)

Diabetes	0,036	0,164	(–0.28, 0.35)	0,335	0,190	(–0.03, 0.70)	**0,425**	**0,184**	(**0.06, 0.78**)	**0,279**	**0,099**	(**0.08, 0.47**)	**0,377**	**0,175**	(**0.03, 0.71**)

Asthma	–0,118	0,132	(–0.37, 0.14)	–0,104	0,156	(–0.40, 0.20)	–0,094	0,146	(–0.38, 0.19)	–0,076	0,079	(–0.23, 0.07)	0,010	0,139	(–0.26, 0.28)

Chronic bronchitis	–0,116	0,216	(–0.54, 0.30)	0,023	0,249	(–0.46, 0.51)	0,421	0,237	(–0.04, 0.88)	0,079	0,127	(–0.17, 0.32)	0,178	0,226	(–0.26, 0.62)

Depression	–0,085	0,141	(–0.36, 0.19)	–0,012	0,164	(–0.33, 0.31)	**0,336**	**0,157**	(**0.02, 0.64**)	**0,211**	**0,084**	(**0.04, 0.37**)	0,236	0,149	(–0.05, 0.52)

Anxiety	0,125	0,120	(–0.11, 0.36)	0,146	0,142	(–0.13, 0.42)	–0,023	0,134	(–0.28, 0.24)	**–0,166**	**0,072**	(**–0.30, –0.0**)	–0,231	0,128	(–0.48, 0.01)

Other mental health disorder	0,314	0,235	(–0.14, 0.77)	–0,018	0,268	(–0,54)–(0,50)	–0,174	0,260	(–0.68, 0.33)	0,013	0,139	(–0.26, 0.28)	0,172	0,247	(–0.31, 0.65)

	Adjusted R2:		0,485	Adjusted R2:		0,498	Adjusted R2:		0,551	Adjusted R2:		0,676	Adjusted R2:		0,496


All significant results are indicated in bold.

Regarding individual characteristics, our results showed that an increase in mental health functioning was associated with significant increases on every dimension of care experience. An increase in physical health functioning was associated with a higher score in accessibility and comprehensiveness. Furthermore, our results showed that men had a more significant score increase on continuity, comprehensiveness and outcome of care than women. It was also observed that patients with diabetes had a significant increase in their score for comprehensiveness, continuity as well as responsiveness while patients with depression noted a significant increase of comprehensiveness and responsiveness. Conversely, patients with anxiety observed a reduction of experience of care in terms of responsiveness between t0 and t2.

## Discussion

Our results showed an overall increase in the care experience of patients who have an IPCT as their primary source of care. To our knowledge, previous studies on patient-reported experience have measured experience of care using cross-sectional designs. QUALICOPC (Quality and Costs of Primary Care in Europe), a study co-funded by the European Commission (EC) aimed at exploring primary health care systems across 34 countries, measured patients’ experiences at first contact with a GP [31]. While providing a comprehensive overview of patient experiences across several countries, changes in patients’ perceptions could not be captured. Another study measuring patients’ experience after receiving care from a nurse practitioner reported a perceived improvement in accessibility to health care providers [32]. The added value of using a longitudinal design is that it allowed us to measure the impact of IPCTs on the care experience over time.

Overall, our results suggest that the four defining characteristics of IPCTs (intensive interdisciplinary practice; advanced nursing practice with an expanded role; group practice; increased proximity and availability) have a positive influence on the patient care experience. Recent literature supports the positive effects of these characteristics on different dimensions of care experience. First, regarding intensive interdisciplinary practice, a review of systematic reviews [33] reported that collaboration between physicians and nurses was associated with better results on blood pressure management, patient satisfaction and hospitalization. The systematic review by Wranik et al. (2019) [9] highlights strong evidence of a positive impact of interdisciplinary primary care on the appropriateness of care and the range of available services. We suggest that the significant increases observed in most IPCTs for the comprehensiveness dimension, as it reflects the scope of services, may be due to interdisciplinary practice.

As for the expanded roles, a 2018 systematic review on nurses working as substitutes for doctors showed that nurse-led primary care may show higher patient satisfaction and may as well lead to higher quality of life for patients [34]. Similarly, in a 2017 scoping review aiming at exploring the work of nurse practitioners in primary healthcare settings [35], it was shown that 13 included studies observed an improvement of self-reported health within nurse practitioner interventions. Our findings support these positive effects, as the only nurse-led clinic of the IPCTs showed an increase of all dimensions of care experience at t2.

As for group practice, collaborative components involving interdependence, collective ownership of goals, role flexibility and shared consultation have been reported to either improve or maintain patient-related outcomes [36]. Furthermore, the review of Shi [37] reported that team-based practice promotes patient-reported relational and informational continuity. The dimension of continuity, which encompassed both relational and management, showed variable scores and very little change over time in all IPCTs. We suggest, therefore, that group practice may be complex and less obviously reflected in care experience.

Finally, regarding increased proximity and availability, a 2017 systematic review community-based nurse-led clinics which are defined as clinics “focused on maintaining people in their communities, and keeping them out of hospital where possible” showed that such clinics have positive effects on patient experience and on patient-reported outcomes as well as an improvement on access [38]. The Increased scores in care experience observed in IPCT E, which is a mobile clinic with several service points, suggest that increased proximity allows for more accessibility and responsiveness.

As we had anticipated, we observed certain differences in patients’ experience of care according to the characteristics of each IPCT. Three of the IPCTs (A, B, C) serve a general population and have a structure similar to common Family Medicine Groups (FMGs), which represents the primary practice model in Quebec. A study using the same methodological approach as our study with the QUALICOPC data from Quebec concluded that the implementation of FMGs has led to slight improvements in accessibility of care and responsiveness while no changes were observed for continuity, comprehensiveness and care outcomes [24]. Our results for IPCTs A, B and C showed that patients for whom these IPCTs have been their primary source of health care two years prior to t0 (Q2) had high scores for all dimensions of experience of care, and that those scores were maintained between t0 and t2. Moreover, we observed score increases for most attributes for Q1 patients from t0 to t2, suggesting that patients who begin to receive care by these IPCTs have an improvement in their experience of care over time. We suppose that the four characteristics that differentiate these IPCTs from the common FMGs may have contributed to these positive results on experience of care.

We observed the most important score increases in IPCTs E and F, which both target vulnerable populations. These increases could be explained by the fact that prior to having one of these IPCTs as their regular source of care, most of the patients from these sites had multiple unmet care needs and received few care. For example, we observed that, for patients from the IPCTs that were implemented less than 2 years before t0, significant score increases were observed on 4 or 5 dimensions. The only dimension of care experience for which a significant increase was not observed for the IPCT E is that of continuity. We suggest that this could be explained by the mission of this IPCT which address all the patients’ needs but gradually reorient them towards conventional care.

We observed a very significant increased score of accessibility in IPCT F. IPCT F focuses on accessible care for vulnerable populations such as patients with HIV/AIDS and Hepatitis C as well as residents of the area with access problems to the healthcare system. The patients have additional concerns which may impact their access to care and, ultimately, to treatments [39]. These concerns include fear of being denied care due to their condition [39]. Offering services that are more aligned with patient preferences and with shared decision-making process would address the needs of this population. We believe this score was achieved due to the fact that the mission of this IPCT places emphasis on accessibility as well as comprehensiveness. Similar results were found in comparable contexts. For example, an Australian study showed that a primary health care clinic for homeless men implemented inside a shelter helped to overcome barriers to health service use and ultimately eased the access to healthcare [40]. Results from this IPCT therefore suggest that this model of care has a significant impact on the care experience of vulnerable populations with high needs of care and limited access to healthcare.

Our findings also showed that certain patients characteristics are associated with higher improvements on the experience of care. Patients with above average mental health status notably reported an increase on every dimension of their experience of care. A 2017 systematic review on determinants of patient satisfaction reported that mental health status was significantly associated with overall patient satisfaction [41]. Absence of mental illness or recovery from anxiety, distress and depression was associated with higher rates of satisfaction of care [41]. The positive results observed in our study regarding the experience of care for these patients therefore suggest that the IPCTs may be well suited to respond to the needs of patients with mental disorders, especially for patients suffering from depression.

Our results also showed that patients with diabetes had an increased experience of care in terms of comprehensiveness, responsiveness and outcomes of care. Studies using the QUALICOPC data showed that a longitudinal and continuous relation with a provider may have a positive effect in stabilizing the condition of patients with diabetes [42]. Moreover, a 2016 study [43] also using the QUALICOPC data observed such effects as continuity of care for patients with diabetes was associated with lower rates of diabetes-related hospitalization. Diabetes mellitus is a complex, multifactorial disease which requires ongoing follow-up by an interprofessional team to prevent the development and progression of complications [44]. In the context where many primary care patients have complex needs [45], it is particularly encouraging to note that the IPCTs with their emphasis on continuity of care and their advanced nursing practice [46] can improve important dimensions of the experience of care for people with more complex long-term health needs such as patients with diabetes.

### Strengths and limitations

Our study is not without limitation. First, as our study was conducted using a quasi-experimental longitudinal design based on a pre-and-post survey without a control group, the lack of random assignment is the major weakness and may have led to threats to its internal validity [47]. Nevertheless, the experience of care questionnaire was very specific to the care received in the IPCTs and it is unlikely that changes in scores reflect a contextual change between the two measurement times. Also, for the second objective, we included in the models many potentially confounding variables. However, we cannot exclude that other potential confounding variables were not measured and were not controlled for. A randomized study could have made it possible to control for these unmeasured or unmeasurable variables [47]. Secondly, the 6 recruited IPCTs were heterogenous and the limited sample (n=6) didn’t allow us to consider and study specific characteristics of the IPCTs and prevented us from performing multi-level analyses. Also, even it was adapted from two validated instruments, the questionnaire used to measure experience of care in this study is not a widely used one. To this day, outside the study from which this tool was designed for [23,48] and subsequent studies using secondary data from this study [24,27], only few studies such as Fournier et al. (2010) [49] used this tool. Because the questionnaire in both rounds of data collection focused on the patient’s past experience, it is possible that some aspects of the care received were missed. However, using the same instrument and setting the data collection interval at two years ensured that patients had little or no recollection of their initial responses and that the measured differences were attributable to their perceptions. Another strength of our study is that we retrieved a large percentage (67.4%) of the original respondents at t2. This high percentage of participants at both collection rounds contributes to the robustness of the data and thus reduces the impact of potential limitations such as possible selection bias.

## Conclusion

Patient-reported measures highlight aspects of care important to the health of populations that are not readily apparent in the standard methods used to assess health system performance. Our results demonstrate that the IPCT model can improve or maintain several dimensions of patients’ care experience. The observed increases in PREMs suggest that this model of practice is tailored to the needs of its target populations. Key differences between IPCTs and standard primary care practice models are a practice mainly consisted of non-physician clinicians, advanced nursing practice with an expanded role, group practice in which teams share resources and responsibilities and increased proximity. These results imply that broader implementation of innovative and flexible community-based care models should be considered by policymakers. This involves rethinking governance, team composition and alignment between the availability of primary care facilities and the needs of populations.

## Additional File

The additional file for this article can be found as follows:

10.5334/ijic.5945.s1 Appendices. Appendix I and II.
