# Supplementary material for: Rethinking Primary Care Delivery Models: Can Integrated Primary Care Teams Improve Care Experience?
Source: Int J Integr Care. 2022 Apr 27;22(2):8. doi: 10.5334/ijic.5945 (PMC9053536; doi:10.5334/ijic.5945)
Supplement: Appendices. — Appendix I and II. [file ijic-22-2-5945-s1.pdf]

Appendix I. Description dimension's items and coding values

Dimension	Item	Description of coding and values (*)
Accessibility	At this place, if the care provider who was responsible for my care was not available, I could see another care provider	Always (10); Often (6,66); Sometimes (3,33); Never (0); NA (Only one professional at this site) (0)
	When you needed to see a care provider at (IPCT), in general, how long did it take to see the care provider by appointment?	Less than 2 weeks (10); From 2 to 4 weeks (6,66); From 1 to 3 months (3,33); 4 months or more (0); I always had pre-scheduled appointment (10); I never took an appointment (10)
	How long does it usually take you to get there?	Less than 15 minutes (10); From 15 to 30 minutes (5); More than 30 minutes (0)
	The office hours were convenient	Agrees (10); Moderately agrees (6,66); Slightly agrees (3,33); Disagrees (0)
	It was easy to reach someone at this place by phone to make an appointment	Agrees (10); Moderately agrees (6,66); Slightly agrees (3,33); Disagrees (0)
	It was easy to talk to a doctor or nurse by telephone when this place was open	Agrees (10); Moderately agrees (6,66); Slightly agrees (3,33); Disagrees (0)
Continuity	When I went to this place, I saw the same care provider	Always (10); Often (6,66); Sometimes (3,33); Never (0); NA (Only one professional at this site) (10)
	My medical history was known (my past medical history)	Agrees (10); Moderately agrees (6,66); Slightly agrees (3,33); Disagrees (0)
	They were aware of all the prescribed medications I took	Agrees (10); Moderately agrees (6,66); Slightly agrees (3,33); Disagrees (0)
	I could receive routine ongoing	Agrees (10); Moderately agrees (6,66);

	care for a chronic problem, for example, for high blood pressure, diabetes, back pain, etc.	Slightly agrees (3,33); Disagrees (0)
Comprehensiveness	All my health problems were taken care of, whether they were physical or psychological	Agrees (10); Moderately agrees (6,66); Slightly agrees (3,33); Disagrees (0)
	During my visits, the health care provider took the time to talk to me about prevention and asked me about my lifestyle habits	Agrees (10); Moderately agrees (6,66); Slightly agrees (3,33); Disagrees (0)
	At the (IPCT), they helped me get all the health care services I needed	Agrees (10); Moderately agrees (6,66); Slightly agrees (3,33); Disagrees (0)
	My opinion and what I want were considered in the care that I received	Agrees (10); Moderately agrees (6,66); Slightly agrees (3,33); Disagrees (0)
	I was given help to weigh the pros and cons when I had to make decisions about my health	Agrees (10); Moderately agrees (6,66); Slightly agrees (3,33); Disagrees (0)
Responsiveness	When you had an appointment at (IPCT), in general, how long did you have to wait between the scheduled time of appointment and the time you actually saw the doctor?	Less than 30 minutes (10); From 30 to 59 minutes (5); 1 hour and more (0)
	Clinic staff answered my questions clearly	Agrees (10); Moderately agrees (6,66); Slightly agrees (3,33); Disagrees (0)
	I felt respected	Agrees (10); Moderately agrees (6,66); Slightly agrees (3,33); Disagrees (0)
	I was greeted courteously at the reception	Agrees (10); Moderately agrees (6,66); Slightly agrees (3,33); Disagrees (0)
	My physical privacy was respected	Agrees (10); Moderately agrees (6,66); Slightly agrees (3,33); Disagrees (0)
	The healthcare provider spent	Agrees (10); Moderately agrees (6,66);

	enough time with me	Slightly agrees (3,33); Disagrees (0)
	The setting was enjoyable	Agrees (10); Moderately agrees (6,66); Slightly agrees (3,33); Disagrees (0)
Outcome of care	The services I got there helped me better understand my health problems	Agrees (10); Moderately agrees (6,66); Slightly agrees (3,33); Disagrees (0)
	The services I got there helped me prevent certain health problems before they appeared	Agrees (10); Moderately agrees (6,66); Slightly agrees (3,33); Disagrees (0)
	The services I got there helped me control my health problems	Agrees (10); Moderately agrees (6,66); Slightly agrees (3,33); Disagrees (0)
	The professionals I saw there encouraged me to follow the treatments prescribed	Agrees (10); Moderately agrees (6,66); Slightly agrees (3,33); Disagrees (0)
	The professionals I saw there helped motivate me to adopt good lifestyle habits like quitting smoking, eating better, etc.	Agrees (10); Moderately agrees (6,66); Slightly agrees (3,33); Disagrees (0)

(*) Every item had the "I don't know/I don't remember" responses option. The latter were aggregated to the most neutral value (5).

Appendix II. Socio-demographic characteristics of respondents by IPCT

Socio-demographic and health characteristics of respondents by IPCT (n=1473)

		Total (%)	IPCT A (n=324)	IPCT B (n=385)	IPCT C (n=420)	IPCT D (n=135)	IPCT E (n=89)	IPCT F (n=121)
Sex	Woman	62.7	65.6	66.2	62.9	71.1	44.4	53.8
Age group	Less than 18 years old	7.0	3.5	8.3	8.3	7.4	1.4	7.6
	18-24 years old	4.8	2.9	6.4	3.8	6.7	12.5	0.8
	25-34 years old	11.3	8.7	9.7	8.8	24.4	25.0	10.1
	35-44 years old	18.7	21.2	11.3	17.9	27.4	20.8	23.5
	45-54 years old	15.2	14.1	15.8	16.7	11.9	13.9	12.6
	55-64 years old	22.4	21.9	27.1	20.5	16.3	22.2	28.6
	65 years old and more	20.6	27.7	21.4	24.0	5.9	4.2	16.8
Matrimonial status (+18 years old)	Never legally married (single)	24.8	25.0	24.0	20.9	39.0	62.3	57.3
	Legally married (and not separated), or law partner	63.4	59.2	65.9	70.4	53.4	23.2	30.8
	Divorced or separated	8.3	11.0	5.7	6.0	4.2	13.0	12.0
	Widowed	3.5	4.8	4.4	2.6	3.4	1.4	0
Highest level of education (+18 years old)	High school diploma or less	28.3	20.5	33.5	30.9	39.7	63.4	40.3
	Trade/vocational school or collegial degree	32.7	23.5	35.9	36.8	19.1	22.5	28.6
	University degree	39.0	56.0	30.5	32.3	41.2	14.1	31.1
Principal occupation (+18 years old)	Full-time worker	45.1	48.6	43.6	42.8	39.8	17.4	41.5
	Part-time work	7.5	6.3	8.9	8.0	13.0	8.5	10.0
	Other	24.1	18.8	23.7	24.6	46.0	65.2	39.8
	Retired	22.1	25.0	26.4	23.7	6.2	4.3	10.2
Perception of economic situation	Financially comfortable	29.4	38.1	33.9	33.1	16.3	4.4	12.1
	Income are sufficient	50.9	49.3	54.1	55.0	44.9	26.5	42.1
	Poor	15.8	9.7	10.8	9.0	32.7	52.9	35.5
	Very poor	3.9	2.9	1.1	2.8	6.1	16.2	10.3
SF-12 physical health score (mean)		48.9	48.8	47.8	50.3	49.4	46.7	47.7
SF-12 mental health score (mean)		48.2	48.3	49.6	49.7	45.5	41.8	47.4
Reported chronic disease	Hypertension	22.9	23.5	25.7	23.6	17.0	16.7	26.9
	Diabetes	9.2	10.9	7.8	9.8	11.9	4.2	9.2
	Asthma	15.7	16.1	17.4	13.3	9.6	20.8	21.8
	Chronic bronchitis	6.0	6.4	4.8	4.8	0.7	20.8	7.6
	Depression	15.8	14.1	13.9	13.8	11.9	33.3	25.2
	Anxiety	23.6	23.8	18.2	21.7	18.5	50.0	33.6
	Other mental health disorder	5.4	2.9	3.8	1.9	6.7	31.9	10.1

[a] Only valid answers are presented.

[b] Due to rounding, the total percentages may be slightly less than or greater than 100%.
